# Is there such a thing as biocompatible peritoneal dialysis fluid?

**DOI:** 10.1007/s00467-016-3461-y

**Published:** 2016-10-08

**Authors:** Claus Peter Schmitt, Christoph Aufricht

**Affiliations:** 1 Pediatric Nephrology, Center for Pediatric and Adolescent Medicine, Im Neuenheimer Feld 430, 69120 Heidelberg, Germany; 2 Division of Pediatric Nephrology and Gastroenterology, Department of Pediatrics and Adolescent Medicine, Medical University of Vienna, Währinger Gürtel 18-20, 1090 Vienna, Austria

**Keywords:** Peritoneal dialysis, Biocompatibility, Glucose degradation product, Icodextrin, Alanyl-glutamine

## Abstract

Introduction of the so-called biocompatible peritoneal dialysis (PD) fluids was based on a large body of experimental evidence and various clinical trials suggesting important clinical benefits. Of these, until now, only preservation of residual renal function—likely due to lower glucose degradation product load and, in case of icodextrin, improved fluid and blood pressure control—have consistently been proven, whereas the impact on important clinical endpoints such as infectious complications, preservation of PD membrane transport function, and patient outcome, are still debated. In view of the high morbidity and mortality rates of PD patients, novel approaches are warranted and comprise the search for alternative osmotic agents and enrichment of PD fluids with specific pharmacologic agents, such as alanyl-glutamine, potentially counteracting local but also systemic sequelae of uremia and PD.

## Medical need: What happens to the patient on PD in 2016?

Treatment with peritoneal dialysis (PD) allows home-based renal replacement therapy (RRT) and thus better patient integration into normal social life, such as school and other aspects important for quality of life (QoL), even though imposing a major burden to families. In pediatric nephrology, PD is the most frequently used dialysis modality until adolescence [1]. More than 90 % of young children are treated with PD as RRT within the first 2 years of life [2]. This is exactly the opposite distribution to adult patients on dialysis, in whom <10 % are treated by PD worldwide, despite improving outcome [3]. Whereas data on morbidity and mortality comparing different forms of dialysis in pediatrics are scarce, such data in adults mostly show comparable outcome, depending on population selection and cultural and socioeconomic background of the healthcare system in the countries in which the studies where performed. Latest analyses of adult patients on RRT even suggest a superior outcome of PD compared with hemodialysis (HD) but a strikingly declining PD usage, at least in Europe [3]. Moreover, studies in the adult population show consistent advantages of PD with regard to QoL indices and economic costs to healthcare providers [4, 5]. However, despite technological progress allowing an acceptable QoL for children waiting for renal transplantation [6], PD treatment is still frequently hampered by infectious and noninfectious complications. A recent large competitive risk analysis in almost 9,000 elderly patients, of whom 50 % required assistance at home, demonstrated that 25 % experienced at least one episode of peritonitis within 1 year. Even without having experienced peritonitis, within 2 years, 11 % of PD patients switched to HD, and >20 % of PD patients died [7]. The individual impact of PD on mortality is difficult to interpret due to the multimorbidity of these cohorts. In pediatrics, data from [2] demonstrated that 20 % of children who were started on PD within the first year of life and who had an array of comorbid conditions died within the first 2 years of treatment, mainly because of infections [2]. Overall, survival of pediatric patients on PD is 94 % at 5 years in countries with a per-capita gross national income above US $28,000 per year and 89 % in countries with lower income [8]. Thus, survival of children with chronic renal failure who end on PD therapy is no better than in children with common oncologic diagnoses [9]. Infectious complication and hospitalization rates declined over the past 30 years in children on PD but are still two- and three-fold higher than in children on HD and after kidney transplantation, respectively [10]. The International Pediatric Hemodialysis Network (IPHN; www.pedpd.org) has monitored children on HD for 3 years and will allow respective comparisons with children on PD. Taken together, there is a high medical need to improve the clinical outcome of children who undergo PD. Current clinical and experimental data suggest that the nonphysiological composition of PD fluids plays a major role in putting the child at risk for infectious and noninfectious complications. This review first focuses on the current status of PD fluids then discusses underlying pathomechanisms that put patients at risk for peritoneal and systemic complications and, second, provides a short summary and outlook on current research and development to tackle these problems in order to improve PD therapy.

## Current PD solutions

The indication for PD fluids is replacement of renal function in acute and chronic renal failure, thus the removal of water and solutes (including uremic toxins) from the uremic patient. The therapeutic effects of these fluids are achieved through their physicochemical composition, i.e., ultrafiltration (UF) of water by the nonphysiologically increased concentration of osmotically or oncotically active agents (such as glucose, icodextrin or amino acids, resulting in an osmolality of 284 mOsmol/l with 7.5 % icodextrin to 511 mOsmol/l with 4.25 % glucose) and low concentrations or absence of solutes that should be removed (mostly intracellular ions, including potassium and phosphate and multiple metabolic waste products). Currently, registered single-chamber-bag glucose-based PD fluids are CAPD®, Dianeal®, and Gambrosol®, and the so-called biocompatible multi-chamber-bag fluids are BicaVera®, Balance®, Gambrosol trio®, Physioneal®, and the non-glucose-based fluids Nutrineal® and Extraneal® (details in [16]. Additional bioincompatibility of these fluids is introduced by pharmaceutical production (e.g., with heat sterilization), resulting in glucose degradation products (GDP) and adducts, but also by economic constraints (such as absence of essential nutrients, as would be present in most cell culture media). As a result, PD fluids are bioincompatible (if not frankly cytotoxic), with different profiles depending on the osmolar active compound and packaging [11].

### Composition of PD fluids

**Fig. 1:**
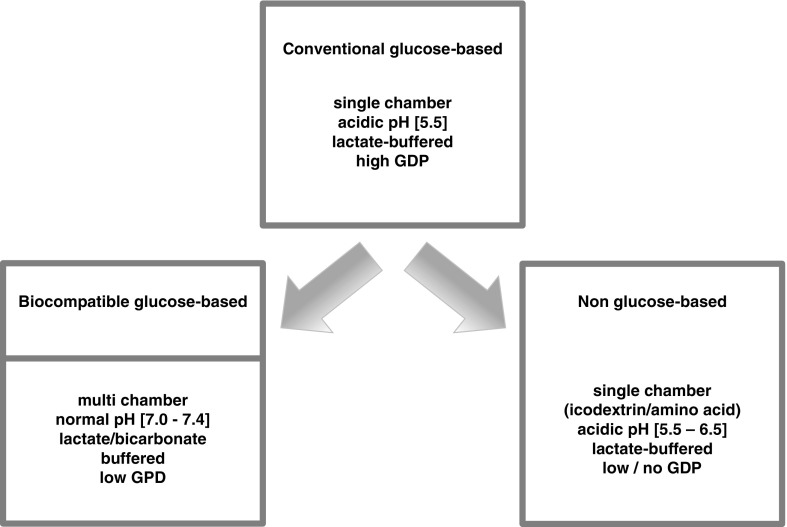
Ontology of peritoneal dialysis (PD) fluids: For ~50 years, conventional PD fluids have been based on glucose as the osmotically active agent and are heat sterilized in single-chamber bags at acidic pH with selected electrolytes (Na, Ca, Mg, and Cl) and a buffer (lactate), resulting in glucose degradation product (GDP) production. In the early 1990s, improved biocompatibility was achieved by introducing multichamber bags to reduce generation of GDP and allow the use of more physiologic buffers (such as bicarbonate) and pH. At the same time, alternative agents were introduced, such as an osmotically active amino acid mixture and the oncotically active glucose polymer icodextrin

Currently, all PD fluids are heat sterilized (Fig. 1). Worldwide, the most abundantly used mixture has remained largely unchanged since its clinical introduction about 50 years ago. In these single-chamber-bags, glucose levels >10–50× normal serum levels are heat sterilized together at acidic pH with selected electrolytes (Na, Ca, Mg, and Cl) and a buffer (lactate). Concentration of the nonphysiological dialysate buffer lactate is 35-fold above normal serum levels; the unphysiologically low pH (5.5) is chosen to reduce generation of highly reactive and cytotoxic GDP and glucose caramelization [12]. This bioincompatibility has been a matter of active research for >30 years and has resulted in more physiological compounding of glucose-based PD fluids in multihamber bags, in which glucose, electrolytes, and buffer substances are separately stored and heat sterilized [13]. This “second generation” of PD fluids allows use of the physiologic buffer bicarbonate at supraphysiological concentrations (34 mmol/l) or at physiological concentration when combined with lactate. Moreover, the separation of glucose and buffer substances allows heat sterilization of glucose at extremely low pH (2–3), resulting in markedly lower—but still biologically relevant—concentrations of GDP and a markedly higher pH (8–9) in the buffer compartment, resulting in neutral to physiological pH of the ready-mixed glucose-based PD fluids prior to clinical use (pH 7–7.4).

In addition, alternative osmotically (or oncotically) active agents were introduced in single-chamber bags, such as the glucose polymer icodextrin and a mixture of amino acids. Albeit reducing the typical toxicity of glucose (heat sterilization of icodextrin still results in glucose degradation byproducts), these novel compounds exhibit nonspecific (such as low pH) and novel substance-specific bioincompatible properties. For example, use of icodextrin resulted in sterile inflammation, most likely due to contamination with bacterial fragments during the production process [14, 15], and an increase in serum levels of icodextrin and its degradation products. Use of amino acids (compounded in PD fluid at about 10× their physiologic serum levels) resulted in different specific perturbances of biological functions, such as vascular effects of arginine [16]. Stable isotope studies in adult continuous ambulatory peritoneal dialysis (CAPD) patients using once-daily amino acid PD solution together with glucose-containing fluid demonstrated improved protein anabolism and a 4 % higher protein synthesis rate compared with patients treated with a glucose-containing PD solution only [17, 18]. According to the International Pediatric Peritoneal Dialysis Network (IPPN) (www.pedpd.org), only 3 % of PD patients use amino-acid-containing fluids, presumably due to the limited anabolic effect and the widespread use of tube feeding.

Low-sodium PD fluids have yielded ambiguous findings in adult PD patients. Applying dialysate solutions with <105 mmol/l of sodium resulted in loss of UF, unless the decline in solution osmolarity was compensated for by glucose [19]. PD fluids with mildly reduced sodium content applied throughout increased dialytic sodium removal by 50 % and reduced blood pressure, but this was accompanied by a decline in residual renal function [20]. Likewise, the concept of adapted automated PD of combining small, short cycles followed by long, large cycles without increasing total dialysate fluid exposure per session might have the potential to improve dialytic sodium removal and blood pressure control and thus to mitigating cumulative PD-fluid-associated toxicity [21]. Mechanisms of the speculated enhanced diffusive transport, however, remain elusive and are not readily explained by the current three-pore model. Suggested benefits need to be balanced against potential risks, e.g., associated with higher intraperitoneal pressure during the large dwells, and require thorough assessment in the pediatric population [22].

### Clinical impact of low GDP PD fluids

With regard to actual outcome data obtained in adequate clinical trials, the level of evidence for improved clinical outcome is still not undisputable in favor of the novel PD fluids. Early clinical trials demonstrated an improved biocompatibility based on effluent markers, such as mesothelial cell viability marker cancer antigen 125 (CA-125) [23, 24], interstitial integrity markers hyaluronan, procollagen-1-C-terminal peptide, and procollagen-3-N-terminal peptide [25], effluent surrogate marker of angiogenesis, vascular endothelial growth factor (VEGF), and of inflammation and interleukin (IL) 6 [26]. Effluent IL6 concentrations predict the highly variable individual peritoneal membrane solute transport function [27]. In a recent Cochrane analysis of clinical benefits of PD solutions, the authors concluded that low GDP fluids do not induce any further harm in PD patients but do not improve the incidence of infectious complications such as peritonitis or preserve long-term peritoneal membrane transport function [28]. Icodextrin solutions improve UF and hydration status. Initially unexpected, the use of two-chamber bags resulted in improved and prolonged maintenance of residual renal urine output and higher residual renal function >12 months of use [28], possibly due to the reduction of GDP-related renal cytotoxicity [29]. However, it can be regarded as a prototype of the level of evidence-based medicine in PD that the largest (and likely best) trial failed in its primary outcome parameter, slowing the rate of glomerular filtration rate (GFR) decline [30]. However, that trial turned out to provide evidence for a protective effect with regard to infectious complications by significantly reducing the incidence of nonpseudomonas Gram-negative peritonitis episodes and the overall severity of peritonitis episodes [31]. Based on these findings, cost effectiveness of low GDP fluid usage has been suggested [32]. Thus, current outcome data in adults still corroborate the notion of the European Pediatric Dialysis Working Group (EPDWG), published in this journal in 2011, that low GDP PD fluids should be used whenever possible [11]. This recommendation was based on randomized controlled trials (RCTs) in adult PD patients [23] and on crossover trials in children with low versus high GDP solutions, demonstrating similar peritoneal solute and water transport kinetics [24], reduced systemic GDP and advanced glycation end-product load [33], superior correction of metabolic acidosis, and higher effluent concentrations of mesothelial cell viability surrogate marker CA125 with the pure bicarbonate-based, low-GDP fluid [34]. Since then, some new clinical data have been reported in the pediatric population. A prospective randomized comparison of low GDP fluids revealed better preservation of UF capacity with bicarbonate versus lactate buffer [35]. Moreover, prospectively collected data in the IPPN suggests improved longitudinal growth in infants treated with low GDP fluids, potentially due to attenuated local and systemic inflammatory and carbonyl stress. This association prevailed even after correction for nutritional status and region but, however, does not rule out confounding factors such as specific socioeconomic status and quality of care associated with low and high GDP fluid usage within the four regions analyzed, and thus requires randomized controlled trials (RCTs) for verification [36].

The impact of low GDP fluids on preservation of long-term peritoneal membrane transport function, however, remains elusive. Small-sized RCTs in adults found no differences in peritoneal transport capacity with low and high GDP fluids over time [37, 38], with the exception of UF capacity in the Euro-Balance trial, in which it declined with pure lactate-buffered low GDP fluid but was fully compensated for by a concomitant increase in residual urine production [23]. In the Balanz trial [39], patients with low GDP fluid usage initially had significantly higher peritoneal solute transport rates, which remained stable over the 2-year follow-up period, and peritoneal UF was lower but increased significantly over time. Patients receiving conventional PD solutions experienced progressive increases in peritoneal solute transport rates and stable peritoneal UF over time. Adequately powered RCTs primarily addressing PD-fluid-induced changes in peritoneal membrane function over time are warranted.

**Fig. 2:**
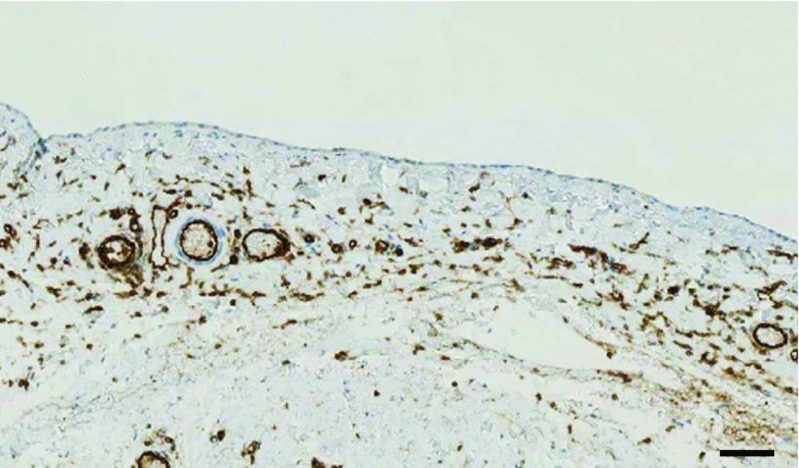
Representative section of the peritoneum of a 13.5-year-old girl on chronic peritoneal dialysis (CPD) devoid of peritonitis. The mesothelial cell layer is still preserved; the submesothelial vessel density [*brown cluster of differentiation (CD31) staining*], however, is markedly increased despite low glucose degradation product (GDP) fluid usage. The three-layer structure of vessels as reported in healthy controls [43] has disappeared (*scale bar* = 100 μm)

Compelling evidence for a persistently high degree of bioincompatibility of low GDP fluids and thus the still great medical need to improve PD fluids to be used in the pediatric population is emerging from the International Pediatric Peritoneal Biopsy Study Group (see Fig. 2). Data from 110 children (85 % on low GDP fluid) obtained in a global effort and analyzed within the European Training and Research in Peritoneal Dialysis (EuTRiPD) consortium (www.eutripd.eu) demonstrate major transformation of the peritoneal membrane with low GDP fluids, including inflammation, progressive fibrosis and angiogenesis [40], i.e. alterations as previously described with high GDP fluids in adults [41], and clearly exceeding uremia-related alterations reported in adults [42]. Quantitative assessment of the healthy parietal peritoneum and omentum in >100 individuals revealed marked age-dependent variations in blood- and lymphatic-vessel density, and derived reference ranges now provide a framework for future histomorphometric analyses and peritoneal transport modeling [43].

## Pathomechanisms relevant in PD

**Fig. 3:**
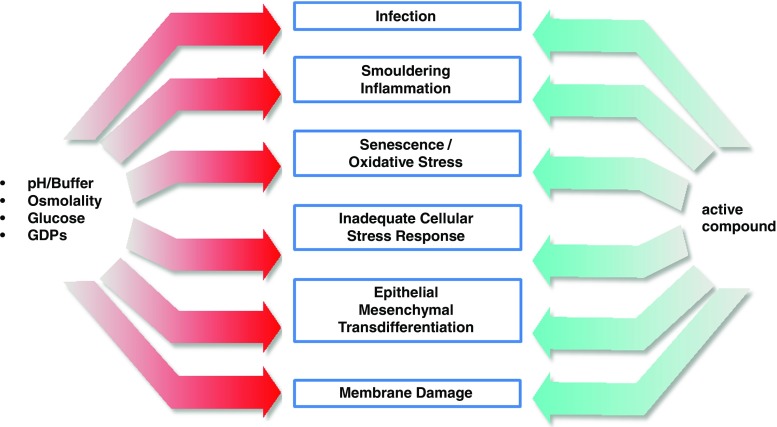
Combination of bioincompatibility of current peritoneal dialysis (PD) fluids in combination with infection and chronic inflammation results in abnormal peritoneal repair processes. Oxidative stress, senescence, and inadequate cellular stress responses are pathomechanisms that mediate glucose-related toxicity, enhance chronic inflammation, and reduce peritoneal host defence, thereby contributing to a vicious circle. Epithelial mesenchymal transdifferentiation (EMT) further mediates peritoneal membrane damage by peritoneal fibrosis and angiogenesis, ultimately resulting in technical failure of PD. These pathomechanisms might be amenable to therapeutic interventions by specific counteracting compounds

PD fluids play a major role in triggering and/or propagating pathomechanisms that are likely relevant for local and systemic complications in PD (see Fig. 3). These complications can be categorized into local and systemic and into infectious and noninfectious complications.

Local infectious complications—in particular, peritonitis—still belong to the most frequent, debilitating, and costly complications of PD [10]. There is a broad body of clinical and experimental data demonstrating that, in particular, heat-sterilized glucose-based dialysis fluids are detrimental for peritoneal host defense [44]. Initial studies focusing on in vitro exposure of different cells of the peritoneal immune system to glucose-based, single-chamber-bag, acidic, lactate-buffered PD fluids provided clear evidence for severe functional impairment and even cell death following more extended exposure [45]. Earlier studies used unspecific cytotoxicity assays, whereas later studies increasingly focused on the role of glucose-related impairment of cells relevant in peritoneal immune defense [12]. Introduction of the multi-chamber-bag PD fluids has markedly reduced the level of acute cytotoxicity; still, these PD fluids are heat sterilized and contain glucose and GDP at concentrations beyond those that were reported in diabetes patients [46]. Metabolic stress by nonphysiological high-glucose levels induces cellular signaling systems (also) involved in immune defense, thereby increasing the risk of peritoneal infection despite reduced amounts of GDP [47]. Currently marketed alternate osmotic agents also appear to result in relatively impaired peritoneal host defense [48]. Thus, intermittent exposure of the immune-compromised peritoneal cavity with bacterial, viral, or fungal species via touch contamination or transintestinal migration still frequently causes subclinical and clinical inflammatory episodes. At present, peritonitis results in technique failure in 8 % of children on PD and in lethal outcome in 1 % [49].

Infectious stimuli together with repeated physicochemical insults by bioincompatible PD fluids propagate smouldering inflammation in the peritoneal cavity. Experimental research in animal and cell culture systems has established a clear role for IL6 in chronic smouldering inflammation triggered by infectious stimuli [50]. IL6 is not only associated with changes in peritoneal transport characteristics, likely reflecting peritoneal remodeling, but also—when measured systemically—with overall outcome and patient mortality [51]. More recently, the IL17 system has been identified as a further important player in peritoneal inflammation [52–54].

The combination of chronic infectious, inflammatory, and cytotoxic insults—in particular, in the context of a diabetes-like milieu—results in abnormal peritoneal repair and stress processes. In particular, regulation of epithelial mesenchymal transdifferentiation (EMT) has been shown to play a major role in peritoneal fibrosis and angiogenesis, ultimately resulting in UF failure in PD [55]. Regarding how far EMT merely represents a good biomarker by which to monitor PD patient management, or whether EMT is an independent pathomechanism that might be amenable for specific interventions, is an ongoing scientific debate [56]. In addition, oxidative stress and senescence are pathomechanisms that likely mediate glucose-related toxicity, in particular glycation processes by reactive carbonyl compounds, resulting in advanced glycosylation end products (AGEs) [57]. Finally, PD fluids result in abnormal cellular stress responses, with dampening of heat-shock proteins, and increased vulnerability of peritoneal cell populations and thereby in enhanced chronic inflammation, peritoneal membrane damage, and hampered host defense [58–60] .

Ultimately, the above described pathomechanisms might not be relevant for local infectious and noninfectious complications only but also for systemic effects, such as further propagating uremia-induced systemic inflammation. Indeed, use of PD fluids that are more biocompatible has resulted in some evidence of reduced systemic inflammation [61] and better maintained residual renal function [28].

## Future solutions

Based on the concept that the common denominator for most pathomechanisms underlying local and systemic complications of PD is glucose-related toxicity, two PD-fluid-related work streams to improve PD therapy become obvious: replacement of glucose by alternative, biologically inert, but osmotic active substances; and/or addition of active compounds to counteract glucose-related toxicity.

The main problem in replacing glucose in PD fluids comes from the massive total daily amount of any such osmotic compound needed. Provided an average prescription of four 2-l bags of 1.5 % glucose-based PD fluid to an adult patient, the local peritoneal exposure amounts to 120 g of glucose, of which more than half is systemically absorbed. Replacement of glucose by amino acids is therefore limited to a single exchange per day due to specific adverse effects, such as elevation of blood urea nitrogen, metabolic acidosis, and uremic symptoms [62]. Absorption of the osmotic agent can only be reduced by using larger compounds, such as icodextrin, but still results in potentially relevant systemic loads [63]. Taken together, the daily absorbed amount of any small/low molecular weight solute from PD fluid needs careful consideration of eventual systemic intolerability, especially in this population with virtually nonexistent renal clearance. Although in experimental PD systems several compounds with attractive biochemical profiles have been successfully applied to replace glucose, only very few of these were ultimately tested in PD patients, and none of them has been approved for clinical use to date. Recently, the glucose-replacing sweetener, stevioside, has been patented for PD use. Hyperbranched polyglycerol has experimentally been introduced as a non-glucose-based osmotic agent in PD fluid and showed promising data in early animal studies [64]. However, due to the above-mentioned need to exclude long-term systemic toxicity of these compounds, administered at doses far above the naturally occurring exposure levels in the vulnerable PD population, extensive safety studies are required prior to their introduction into clinical practice. Therefore, the use of clinically available alternative solids is currently limited to icodextrin and an amino acid mix dosed as a single bag per day.

Due to the relative safety of glucose experienced over >50 years of intraperitoneal use and its well-known side effects, an alternative approach is the addition of active compounds counteracting glucose-related toxicity (see Fig. 3). The majority of these compounds (as reviewed in detail in [65] and in [66]) have been selected by a hypothesis-driven approach. Several of these compounds are highly specific pharmacologic agents or biologics that counteract specific pathways identified to be relevant in PD-related complications. For example, peroxisome proliferator-activated receptor (PPAR)-gamma agonists, such as rosiglitazone, have been shown in experimental PD systems to result in improved membrane integrity and to reduce inflammation. Addition of bone morphogenic protein (BMP)-7 mitigates mechanisms such as chronic inflammation and EMT (reviewed in detail in [65], [66]). More recently, use of antagomir has proven to effectively counteract peritoneal fibrosis in a rodent model of PD [67]. However, similar to the case with complete replacement of glucose, these specific medications will need thorough preclinical and clinical testing before the possibility of their clinical use. According to www.clinicaltrials.gov, of these compounds, only glycosaminoglycans such as heparin and low molecular weight heparinoids have reached the clinical stage but have not resulted in reproducible clinical benefits (online search January 2016).

Another promising approach is the addition of nontoxic agents to PD fluids in doses shown to be safe by prior use as nutritional supplements in patients with stage 5 chronic kidney disease on dialysis (CKD5D). Historically, several such compounds were tested in experimental settings but did not successfully undergo further clinical development (http://www.uptodate.com/contents/peritoneal-dialysis-solutions). Of these compounds, only carnitine and alanyl-glutamine dipeptide are in clinical development. For both agents, favorable profiles of systemic effects have been previously reported, such as improved metabolism and immune competence [68]. In a recent phase II trial in nondiabetic PD patients, carnitine-enriched PD fluids improved glucose metabolism [69]. Addition of alanyl-glutamine has resulted in restoration of cellular stress responses, attenuation of peritoneal inflammation, and protection of the peritoneal membrane in experimental systems [54, 60] and is being tested in a phase II trial (EudraCT Number: 2013-000400-42). It certainly remains to be proven in prospective phase III trials whether novel “third-generation PD fluids” are able to prevent the complications that currently hamper PD treatment.

## Perspectives

A large body of experimental evidence and respective RCTs have resulted in the introduction of the currently available, so-called biocompatible, PD fluids, which have yielded certain clinical benefits such as better preservation of residual renal function and, in the case of icodextrin, improved fluid control and blood pressure. However, they still exert considerable toxicity, and their benefits regarding infectious complications, PD membrane preservation, cardiovascular health, and patient survival still need demonstration. PD patient morbidity and mortality is still unacceptably high in both adult and pediatric patients, mandating innovative approaches, i.e., PD fluids with less local and systemic toxicity and, ideally, even actively counteracting uremic sequelae such as carbonyl and oxidative stress, as may be the case with specific pharmacologic agents such as alanyl-glutamine dipeptide. Introduction of various novel PD fluids with specific local and systemic benefits should allow for a more personalized treatment of PD patients. Not all patients may benefit from these more costly PD fluids, and thus identification of those who will have a particularly high benefit from the novel therapy or who have a particularly high risk with the current standard fluids is required. Thus, development of new PD fluids must go hand-in-hand with the development of biomarkers to identify patients in need of these innovative therapies. How far this approach will improve overall outcome and QoL of PD patients will be the topic of future research.
